# Towards Providing Effective Data-Driven Responses to Predict the Covid-19 in São Paulo and Brazil

**DOI:** 10.3390/s21020540

**Published:** 2021-01-13

**Authors:** Fabio Amaral, Wallace Casaca, Cassio M. Oishi, José A. Cuminato

**Affiliations:** 1 Faculty of Science and Technology, São Paulo State University (UNESP), Presidente Prudente 19060-900, Brazil; fabio.amaral@unesp.br (F.A.); cassio.oishi@unesp.br (C.M.O.); 2 Department of Energy Engineering, São Paulo State University (UNESP), Rosana 19273-000, Brazil; 3 Institute of Mathematics and Computer Sciences, University of São Paulo (USP), São Carlos 13566-590, Brazil; jacumina@icmc.usp.br

**Keywords:** Covid-19, SIRD, data-driven models, machine learning, interactive platform

## Abstract

São Paulo is the most populous state in Brazil, home to around 22% of the country’s population. The total number of Covid-19-infected people in São Paulo has reached more than 1 million, while its total death toll stands at 25% of all the country’s fatalities. Joining the Brazilian academia efforts in the fight against Covid-19, in this paper we describe a unified framework for monitoring and forecasting the Covid-19 progress in the state of São Paulo. More specifically, a freely available, online platform to collect and exploit Covid-19 time-series data is presented, supporting decision-makers while still allowing the general public to interact with data from different regions of the state. Moreover, a novel forecasting data-driven method has also been proposed, by combining the so-called Susceptible-Infectious-Recovered-Deceased model with machine learning strategies to better fit the mathematical model’s coefficients for predicting Infections, Recoveries, Deaths, and Viral Reproduction Numbers. We show that the obtained predictor is capable of dealing with badly conditioned data samples while still delivering accurate 10-day predictions. Our integrated computational system can be used for guiding government actions mainly in two basic aspects: real-time data assessment and dynamic predictions of Covid-19 curves for different regions of the state. We extend our analysis and investigation to inspect the virus spreading in Brazil in its regions. Finally, experiments involving the Covid-19 advance in other countries are also given.

## 1. Introduction

According to the official report published by the World Health Organization (WHO) [1], up to November 2020, the novel coronavirus infected more than 6 million people in Brazil. While some countries in Europe are facing the second wave of the pandemic, Brazil is suffering the adverse impacts of Covid-19’s lasting wave while still preparing for the arrival of a new wave hitting the country at the end of the year. In particular, in the state of São Paulo, which is the most populous state, holding around 22% of the country’s population, the infections have reached more than 1 million people [2].

**Figure 1:**
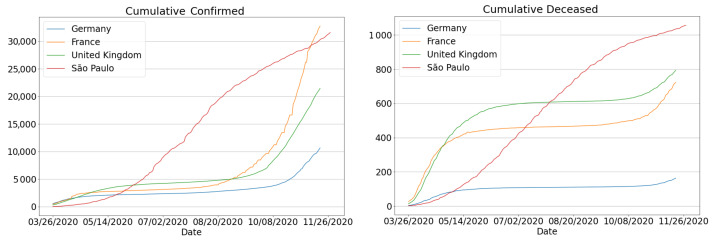
Comparison of cumulative number of cases and deaths per million: São Paulo state, France, Germany and United Kingdom. Country data are from Johns Hopkins University [4].

Another disconcerting fact about the Covid-19 situation in the state of São Paulo is that it currently accounts for 25% of all deaths in the country [2]. As a result, the state has been the epicenter of the coronavirus outbreak in Brazil. It can be compared to other countries, placing São Paulo (until October 2020) in the 5th and 6th positions globally with respect to confirmed cases and deaths, thus above Germany, France and the UK [3], as one can see in Figure 1.

Due to the unclear scenario of the Covid-19 pandemic in São Paulo state, the public health system has been dealing with many challenging issues as those currently faced by other countries such as the availability of free beds in hospitals [5,6], monitoring of control measures [7,8,9] and implementation of immediate mitigation plans [10,11] to contain the advance of coronavirus. These also include the development of effective data-driven responses such as real-time monitoring systems and forecasting models to track and predict Covid-19 advance in each region of the state, even under real-world circumstances that are hard to handle in practice. For example, a drastic reduction in data updates for a few days, because of a delay in making Covid-19 test results public as well as a retroactive data refresh due to inconsistencies that occur when managing multiple data sources, can result in a poorly trained model with high chances of failure when fitting Covid-19 data. Moreover, the forecasts strongly reflect the accuracy of the collected data, as notifications are usually recorded by date of disease confirmation rather than the date of occurrence. In fact, more realistic predictions depend on successive updates with accurate data in order to be effective [12,13,14]. Therefore, the first goal of this work is to address the issue of inaccurate/delayed data for predicting 10-day Covid-19 curves with a satisfactory level of accuracy.

Another issue when extrapolating epidemiological data is that the model’s parameters are assumed to be constant, e.g., transmission rate and rate of recovery, as typically taken by classic Susceptible-Infectious-Recovered (SIR)-based approaches [15,16]. Despite the existence of very effective SIR variants that take the model’s parameters as constant [17], their calibration when concomitantly assessing a great variety of regions with distinct traits is not a straightforward task since some of the tunable values depend on local government-regulated measures, which are difficult to get in practice, especially in the Brazilian context. In order to circumvent the parameter issue of classic SIR-derived methods while still allowing the mathematical model to cope with time-varying coefficients, the use of Machine Learning strategies has been a popular choice and a trend. Indeed, recent developments involving variable-parameter SIR variants to assess the course of Covid-19 can be found in [18,19,20,21,22,23,24,25,26,27,28], which include the use of effective Artificial Intelligence (AI) strategies, for example in [18,19,29,30,31,32,33]. Following these recent efforts in modeling Covid-19 dynamics from epidemic models tuned with learning mechanisms, in this paper we propose an effective, data-driven SIR model whose parameters are fully calibrated by temporal functions, learned from individual regressors and trained on different data sources. The predictions are obtained using a time-dependent SIR-based model [34] coupled with an intelligent architecture that learns the model’s parameters for each one of the regions analyzed in our study.

A important advantage of our data-driven approach is that it only assumes as input the raw data of infected, recovered and deaths to produce the definitive forecasts. In fact, the current learning scheme does not require any prior knowledge of specific time-series such as the transmission rate curve. Another relevant aspect to be observed is that the designed technique does not impose any particular probability distribution to the epidemiological curves, thus avoiding the use of pre-fixed forms of data distribution such as exponential solutions and logistic regression-based models.

Contributions The main contributions of this paper can be summarized as follows:

- The implementation of urgent responses, as listed below, to mitigate the progress of coronavirus in São Paulo state, which is the most populous and economically active state in Brazil, responsible for 34% of the Brazilian GDP [35].
- A novel forecasting model that combines the simplicity of SIR-based formulation with the effectiveness of data-driven learning strategies for predicting Covid-19 cases, deaths, recoveries and the virus reproduction number. The designed method is also capable of addressing “the curse of delay”, as usually observed in the Brazilian reports of cases and deaths, determining whether or not a coronavirus-related time-series period is “well-posed”.
- Our predictive approach learns the epidemiological parameters as time-dependent functions, which are calibrated by a recursive training approach based on an Artificial Neural Network, therefore allowing the forecaster to fit and customize Covid-19 curves for each region of the state.
- The availability of a comprehensive Covid-19 data repository and a freely available online platform, which has been accessed by citizens, authorities and media agencies to track and inspect the Covid-19 progress in São Paulo state. New Covid-19 notifications are immediately available throughout the platform, by getting fresh data published daily by 92 city halls spread over the state (the so-called first-hand local sources), in an attempt to reduce the delay in reporting the new cases and deaths as often observed in the Brazilian government updates [36,37].

This paper is organized as follows: Section 2.1 introduces the problem description and the mathematical design of our data-driven epidemiological model, while Section 2.2 describes the details of the proposed training apparatus to learn the model’s parameters. Next, Section 3 brings the validation study of our approach and numerical experiments with real data focused on São Paulo state and Brazilian regions. Experiments involving other countries are also given. Finally, Section 4 summarizes our findings, observed conclusions and future work, while in the Appendix A, Appendix B,Appendix C, we present our online tracking platform used by decision-makers and other interested people in interacting, auditing and navigating coronavirus-related data in the state of São Paulo. Implementation details as well as side forecast results are also provided as part of the Appendix A, Appendix B,Appendix C.

## 2. Materials and Methods

### 2.1. Mathematical Modeling: A Time-Dependent SIR-Based Model

In this section, we present the mathematical design for the proposed data-driven epidemiological model to forecast Covid-19 trends.

Let *N* be the size of the total population we intend to model. The classical SIR model [34] is given by the following system of Ordinary Differential Equations (ODEs):

(1) $$\begin{array}{cc} \; & \frac{dS}{dt}=-\beta \frac{SI}{N},\\ \; & \frac{dI}{dt}=\beta \frac{SI}{N}-\gamma I,\\ \; & \frac{dR}{dt}=\gamma I, \end{array}$$

where S=S(t), I=I(t) and R=R(t) are the numbers of susceptible, infected and recovered individuals, respectively, as the time *t* varies. The canonical form of SIR modeling assumes N=S(t)+I(t)+R(t), while the transmission rate β and the rate of recovery γ are taken as real constants. The so-called basic reproduction number $R_{0}$, which is one of the key metrics in epidemiology, is defined by $R_{0}=\frac{\beta }{\gamma }$ [21,38].

In our mathematical approach, we introduce a new population group D(t) to represent the total number of infected people who died. A normalized total population, N=1, is also taken in the ODE system (1) so that the resulting modified SIR model, namely Susceptible-Infectious-Recovered-Deceased (SIRD) [39], is derived:

(2) $$\begin{array}{cc} \; & \frac{dS}{dt}=-\beta SI,\\ \; & \frac{dI}{dt}=\beta SI-\left(\gamma_{r}+\gamma_{d}\right)I,\\ \; & \frac{dR}{dt}=\gamma_{r}I,\\ \; & \frac{dD}{dt}=\gamma_{d}I. \end{array}$$

Parameters $\gamma_{r}$ and $\gamma_{d}$ account for the rates of recovered and mortality, respectively. In our formulation, we assume that the transmission rate has a transient trajectory, i.e., β=β(t). As a consequence, we get a time-dependent reproduction number on the form:

(3) $$R_{0}\left(t\right)=\frac{\beta (t)}{\left(\gamma_{r}+\gamma_{d}\right)}S.$$

The so-called effective reproduction number, $R_{0}\left(t\right)$ or $R_{t}$, is an important epidemiological metric that quantifies the average number of new infections arising from a primary infected individual in the population [25,40]. In practice, $R_{t}$ measures the Covid-19 spread rate, and it changes as either the individuals gain immunity or die. The ODE system (2) with β=β(t) is also known as variable coefficient Susceptible-Infected-Removal (vSIR) [21], time-varying SIR epidemic [22], or simply as time-dependent SIR model [19,23]. Table 1 lists the mathematical symbols used in this work.

The Differential Equations system (2) is numerically solved for S,I,R and *D* from a given set of initial condition values, $S_{0}$, $I_{0}$, $R_{0}$ and $D_{0}$, producing the numerical solutions $\bar{S}=S\left(t_{n}\right)$, $\bar{I}=I\left(t_{n}\right)$, $\bar{R}=R\left(t_{n}\right)$ and $\bar{D}=D\left(t_{n}\right)$ for a discretized time $t_{n}$ with a fixed time step Δt. To do so, we run the Livermore Solver of Ordinary Differential Equations with Automatic Method Switching (LSDOA) [41], which is implemented in the Python library scipy. ODE system (2) is recurrently solved as part of an integrated training pipeline, which learns the model’s parameters according to data signatures of each state region, as we will discuss below.

### 2.2. Learning Epidemiological Parameters: An Integrated Data-Driven Approach

**Figure 2:**
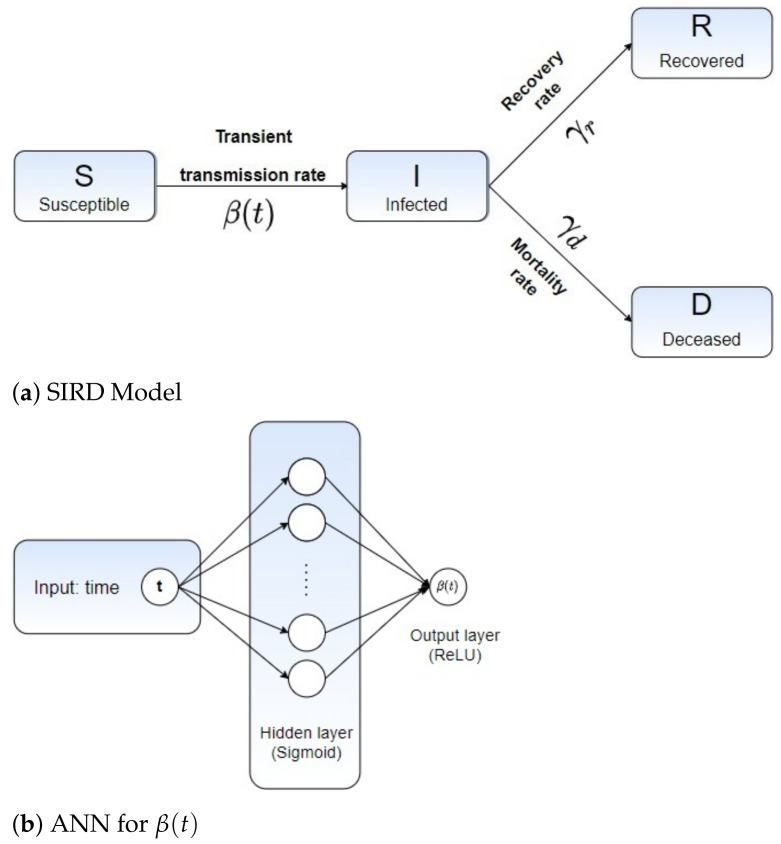
(**a**) Susceptible-Infected-Recovered-Deceased (SIRD) model with its corresponding parameters and (**b**) the ANN design for learning β(t)

In this section, we describe our hybrid machine learning pipeline to fit the epidemiological parameters $\beta \left(t\right),\gamma_{r}$ and $\gamma_{d}$ by recursively refining the solution of the ODE system (2). The proposed learning scheme relies on the solution of an inverse problem, given in terms of the SIRD model (2) coupled with an Artificial Neural Network (ANN) to learn from the Covid-19 data, the infected, recovered and deceased cases, denoted here as $I_{data}$, $R_{data}$ and $D_{data}$. The unified ANN architecture with SIRD model is illustrated in Figure 2.

We construct an ANN to predict the values of β(t) for each discrete time $t_{n}$, generating a full time-varying curve $\beta_{net}\left(t\right)$. The proposed ANN architecture is composed of a hidden layer, containing 10 neurons, and the Sigmoid kernel as the network activation function. The output layer is fully connected to the hidden layer thorough a single neuron with no bias weights, wherein the ReLU is taken to trigger the neuron. As the loss function, we minimize the following aggregated error measure, given in terms of the model’s variables *I*, *R* and *D*:

(4) $$\begin{array}{cc} \; & L\left(\beta_{net}\left(t\right),\gamma_{r},\gamma_{d}\right)=l_{I}+l_{R}+l_{D} \end{array}$$

where:

(5) $$\begin{array}{cc} \; & l_{I}=\frac{1}{M}\sum_{n=0}^{M}{∥log\left(I_{data}\left(t_{n}\right)\right)-log\left(\bar{I}\left(t_{n}\right)\right)∥}_{2}^{2}, \end{array}$$

(6) $$\begin{array}{cc} \; & l_{R}=\frac{1}{M}\sum_{n=0}^{M}{∥log\left(R_{data}\left(t_{n}\right)\right)-log\left(\bar{R}\left(t_{n}\right)\right)∥}_{2}^{2}, \end{array}$$

(7) $$\begin{array}{cc} \; & l_{D}=\frac{1}{M}\sum_{n=0}^{M}{∥log\left(D_{data}\left(t_{n}\right)\right)-log\left(\bar{D}\left(t_{n}\right)\right)∥}_{2}^{2}\;. \end{array}$$

In Equation (5), Equation (67) and Equation (7), *M* is the pre-specified training period, ${∥\cdot ∥}_{2}$ accounts for the euclidean norm, and the log operator has the role of improving training performance regardless of data scalability, as the analyzed dataset is normalized before running the learning process.

The SIRD parameters $\beta_{net}\left(t\right)$, $\gamma_{r}$ and $\gamma_{d}$ are predicted by solving the following ANN-related optimization problem:

(8) $$\underset{W,b,\gamma_{r},\gamma_{d}}{arg min}\;L\left(\beta_{net}\left(t\right),\gamma_{r},\gamma_{d}\right).$$

In the proposed learning formulation, the trained parameters are the set of the ANN weights {W,b}, and the outputs are the time-varying function $\beta_{net}\left(t\right)$ and the epidemiological parameters $\gamma_{r}$ and $\gamma_{d}$. In our tests, we solve the ANN optimization problem (8) by running the Limited Memory Broyden–Fletcher–Goldfarb–Shanno (L-BFGS) algorithm [42]. Notice that the use of ANN instead of any other particular data-driven approach relies on two basic aspects: (i) the effectiveness of neural network design in learning trends and patterns from time-series data, and (ii) the success of more recent works on applying ANN to forecast Covid-19 epidemiological curves as, for example, in [43,44].

**Figure 3:**
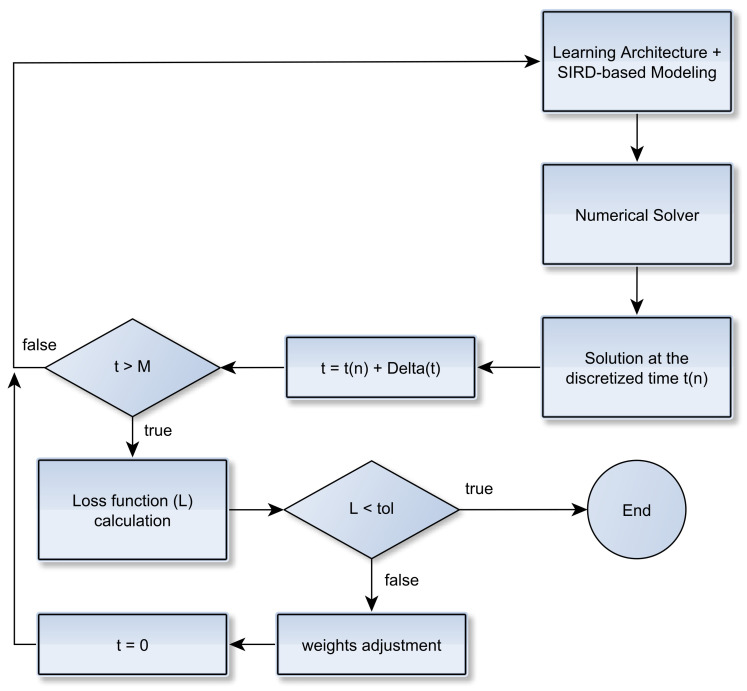
Illustration of the parameter calibration step.

Once the epidemiological parameters are estimated via the neural network architecture, we solve the ODE-SIRD system (2) for the infected, recovered and deceased cases so that a recursive learning procedure is established. More precisely, the loss function $L(\beta_{net}\left(t\right),\gamma_{r},\gamma_{d})$ is re-evaluated for the current set of SIRD parameters, and both the numerical resolution of Equation (2) and the training scheme (8) are repeated until the loss function reaches a minimum. Figure 3 illustrates this step.

#### Improving Data Fitting Robustness and Accuracy

As our pipeline makes use of fresh data to learn the parameters, the untimely posting of a few city datasets in certain time intervals may affect the training task, especially when *M* varies in ODE-SIRD system (2). A low value for *M* may cause the training to ignore past events, thus overfitting the most recent disease occurrences. On the other hand, taking a large value for *M* can lead the mathematical model to drop newer information. Additionally, as the data are recurrently updated, there is no straightforward way to detect these particular badly conditioned sub-intervals over the full time-series.

In order to improve data fitting of ill-behaved data portions while preserving the epidemiological traits of SIRD modeling, we have adopted a moving window-based strategy to balance the contributions for the forecasted variables over different training intervals. More precisely, our approach takes the following steps:

1. Compute training outputs for several time windows by repeatedly solving the ODE-SIRD system (2) for $M=M_{i}\in \left\{M_{1},M_{2},...,M_{n}\right\}$, where $M_{1}=10,M_{2}=11,...,M_{n}=30$ days, calibrating the net weights, bias, and parameters $\gamma_{r}$ and $\gamma_{d}$ for different simulation intervals.
2. Once the set of epidemiological curves $\Lambda =\{C_{i}\;:C_{i}=\left\{I_{i}\left(t\right),D_{i}\left(t\right),R_{i}\left(t\right)\right\}\}$ is obtained, we compute the Mean Absolute Percentage Error (MAPE) (9), taken here as an error assessment metric, to decide whether or not a subset of $C_{i}^{\prime }s$ from Λ is classified as “outlier”, i.e., a badly conditioned time-series period whose epidemiological variables $I_{i}\left(t\right)$, $D_{i}\left(t\right)$, $R_{i}\left(t\right)$ and $R_{0}\left(t\right)$ highly diverge from other periods. In our tests, we discard the ill-behaved $C_{i}$’s whose MAPE errors are greater than 20% for any of the variables $I_{i}\left(t\right)$, $D_{i}\left(t\right)$ or $R_{i}\left(t\right)$.
3. Finally, the remaining trained curves are used to compute the definitive forecasts using the numerical solution of the SIRD system for t∈[0,M+p], where *p* is the desirable forecast period. This is performed so as to balance the well-behaved contributions in the set of ODE solutions Λ, taking the mean of these outputs to determine $I_{i}\left(t\right)$, $D_{i}\left(t\right)$, $R_{i}\left(t\right)$ and $R_{0}\left(t\right)$.

**Figure 4:**
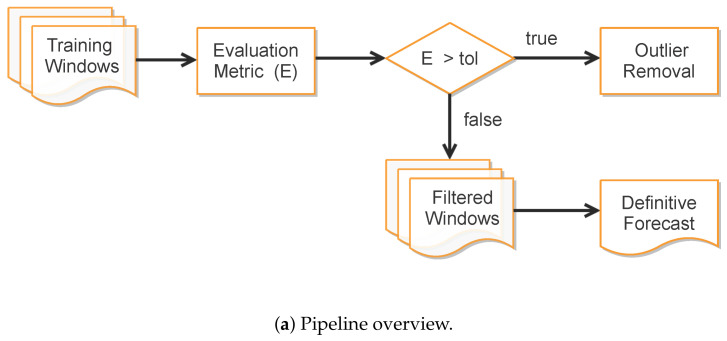

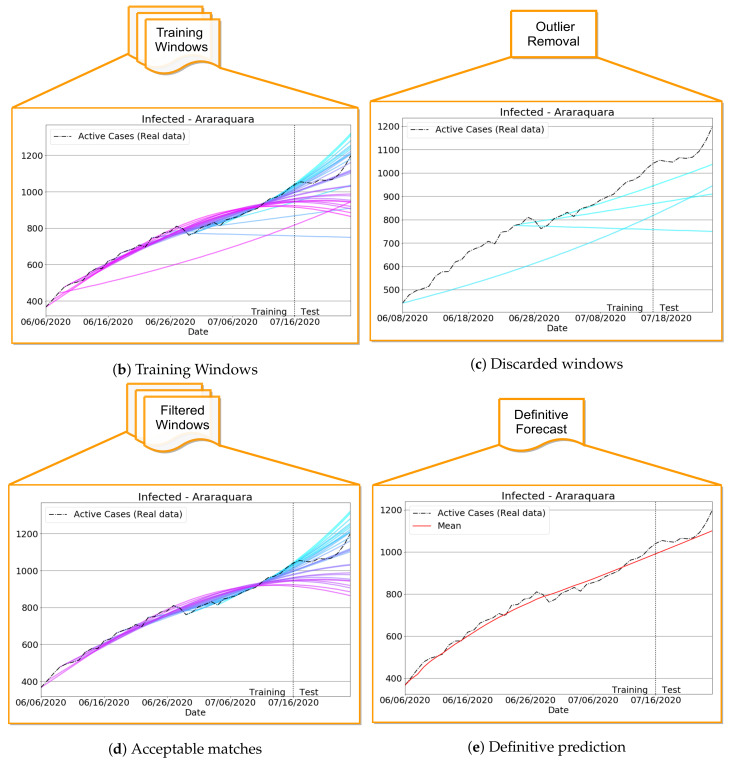
(**a**) The complete filtering pipeline. (**b**) Training outputs for different time windows. (**c**) The selected ill-behaved training periods (discarded trainings). (**d**) Training results that have passed the error criteria for good training. (**e**) Averaged results as the definitive prediction.

The rationale behind the above-described outlier filtering scheme is that it prevents bad training results that interfere with the forecast quality. Indeed, the filtering acts as an adaptive data-driven classifier, identifying badly conditioned time window periods over the full time-series while still ensuring a better data fitting performance and smoothing. Further, as the effective reproduction number $R_{0}\left(t\right)$ drives the slope of the infection curve (if $R_{0}\left(t\right)<1$, the number of new infections in the next generation will be reduced, while $R_{0}\left(t\right)>1$ holds the opposite situation), it is expected that different successful training results produce similar estimations for the true observed data of *I*, *D* and *R* so that the learning process will take into account only well-behaved parameters to estimate the definitive $R_{0}\left(t\right)$ curve. Figure 4 illustrates the filtering approach results, while the implementation details are given in Appendix C.

## 3. Results and Discussion

### 3.1. Data Organization

**Figure 5:**
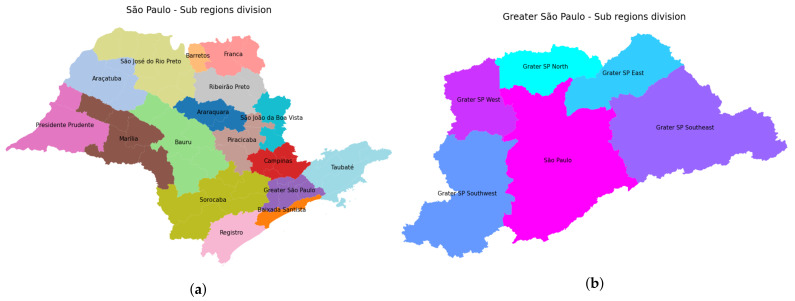
Sub-region maps of São Paulo state: (**a**) State map showing the 22 state sub-regions, and (**b**) São Paulo metropolitan region.

In order to track the daily evolution of Covid-19 while collaborating with the decision-makers of the Brazilian public body, we rearranged the collected data into 22 large regions corresponding to each Regional Health Department of the state (see Figure 5a). Particularly, due to the huge urban sprawl around São Paulo city, state government has grouped the so-called Greater São Paulo Region into seven sub-regions (São Paulo North, São Paulo Southeast, São Paulo Southwest, São Paulo Northeast, São Paulo Metropolitan, São Paulo East and São Paulo West), as illustrated in Figure 5b. As a result, for each one of the 22 Health Departments, time-series for confirmed cases and deaths were obtained, with entries ranging from 1 April to 31 October, i.e., a seven-month period of daily records.

### 3.2. Metrics

In our experiments, the forecasts are assessed by applying the *Mean Absolute Percentage Error* (MAPE), a classic evaluation metric widely used in time-series analysis [45,46]:

(9) $$MAPE\left(Y_{i},\dot{Y_{i}}\right)=\frac{1}{n}\sum_{i=1}^{n}\left|\frac{Y_{i}-\dot{Y_{i}}}{Y_{i}}\right|\times 100,$$

where $Y_{i}$ and $\dot{Y_{i}}$ account for the real and predicted daily values of any target variable as forecasted by our data-driven model. In our assessments, we follow [46] so that a threshold of 10 % is established for MAPE in order to ensure a “satisfactory level” of accuracy regarding the predictive performance.

Another evaluation metric taken in our qualitative analysis is the Normalized Root Mean Square Error (NRMSE), computed according to the following expression [47]:

(10) $$\begin{array}{c} NRMSE\left(Y_{i},\dot{Y_{i}}\right)=\frac{1}{n}\frac{\sqrt{\sum_{i=1}^{n}{\left(Y_{i}-\dot{Y_{i}}\right)}^{2}}}{\bar{Y}}, \end{array}$$

where $\bar{Y}$ determines the average of the observed data.

Finally, we also make use of the Variance to assess how the trained parameters can affect the reproduction number $R_{0}\left(t\right)$ as the training interval *i* in the SIRD model varies. Such statistical metric is calculated for each time $t_{j}$ of the training period by applying the following formula:

(11) $$s_{j}^{2}=\frac{1}{n_{j}-1}\sum_{i=1}^{n_{j}}{\left(R_{0}^{\left(i\right)}\left(t_{j}\right)-\bar{R_{0}}\left(t_{j}\right)\right)}^{2}\;,$$

where $R_{0}^{\left(i\right)}\left(t_{j}\right)$ represents the estimated value of $R_{0}\left(t\right)$ at the discretized time $t_{j}$.

### 3.3. The Proposed Forecasting Approach: Main Features and General Capabilities

#### 3.3.1. Badly Conditioned Samples × Data Fitting Robustness and Accuracy

**Figure 6:**
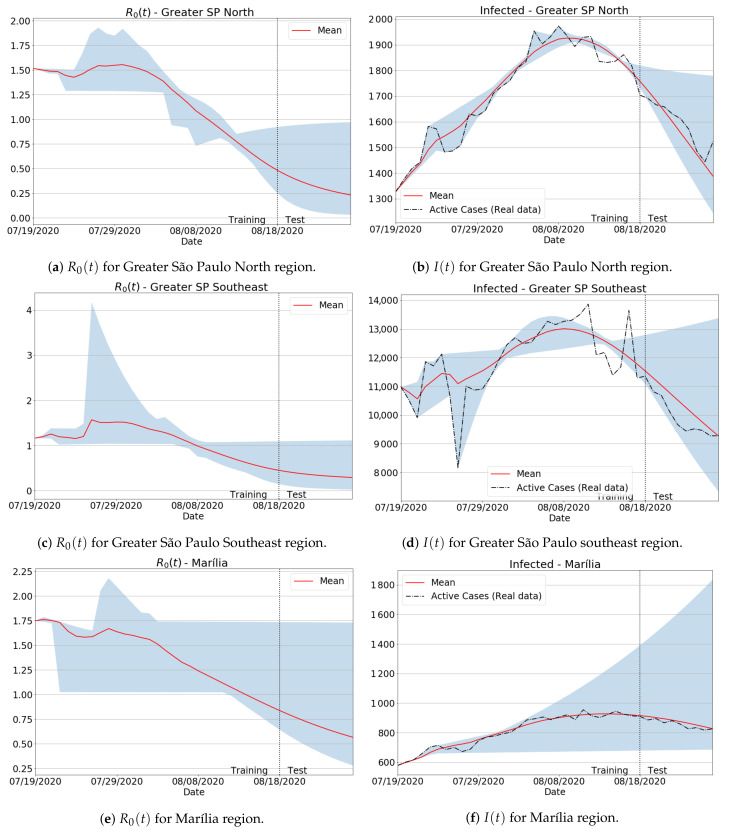

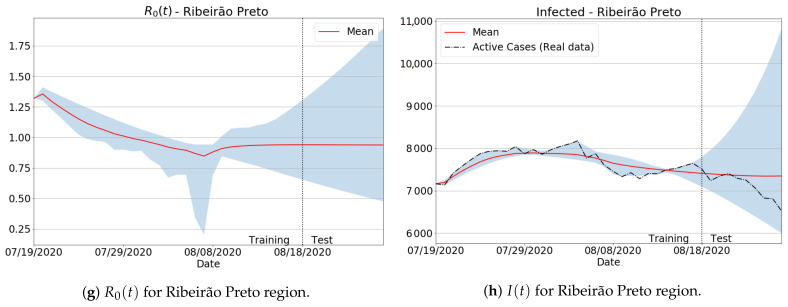
Reproduction number $R_{0}\left(t\right)$ and infected I(t) predictions for the mean, minimum and maximum forecasted values as the training window moves, i.e., by varying M=10,11,…40 in Equation (2) and training the parameters in a coupled and recursive way. Red lines establish the mean predicted values after the full learning procedure is finished, while the vertical dotted lines split the training and forecasting periods.

As previously discussed in Section 2.2, the amount of data used to calibrate the model’s parameters can impact the Covid-19 estimations, such as the actual infections I(t) and reproduction number $R_{0}\left(t\right)$, as specific time-series periods are made up of badly conditioned data. In order to show how such an issue can influence the forecasts, and how our moving window-based training scheme can fix it, we present in Figure 6 both the $R_{0}\left(t\right)$ and infection I(t) curves in the period when there were no full updates of Covid-19 data in several São Paulo state regions, as pointed out by the Brazilian press news [48]. Notice from the results with badly conditioned data that although the predicted values produced large peaks and valleys in both $R_{0}\left(t\right)$ and I(t) curves, the definitive forecasts (in red) were successfully fitted, keeping very close to the true data. Indeed, even in more drastic cases involving bad behaving data (see Greater SP Southeast and Marília regions), our data-fitting approach performed well, ensuring the correct tendency of the real curves. Finally, it can be seen that the reproduction number $R_{0}\left(t\right)$ dictated the slope of infection curves, as expected.

The forecasting results from Figure 6 were also assessed via quality metrics. Besides the well-established MAPE score, we take Equation (11) as a popular assessment metric to gauge data variability and inconsistency level. More precisely, given a fixed point $t_{j}$ in the simulation domain, we compute the variance $s_{j}$ with respect to all *i* samples of $R_{0}^{\left(i\right)}\left(t_{j}\right)$, generated as the i-th training window varies during the full learning process. As a result, if the variance $s_{j}$ is low, the trainable infected values for all *i* indices will follow the same common tendency, which means that the node $t_{j}$ does not hold badly conditioned data. On the other hand, if the variance is high, then there are training intervals *i* probably inconsistent and badly behaved. From Table 2, one can check that our training approach delivered low error measurements, producing very stable estimations with low prediction variations, as measured by the variance. For example, the largest MAPE error was around 4%, while the highest value for the variance to the predicted effective reproduction number $R_{0}\left(t\right)$ was 1.478.

#### 3.3.2. The Transient Behavior of Transmission Rate

As discussed in Section 2.2, an important strategy adopted in our mathematical approach is the use of a simple, but effective, Artificial Neural Network (ANN) for estimating the transmission rate β in a transient context.

To better emphasize the neural network importance in ensuring a transient behavior to the transmission rate, we compared the results with/without the ANN so that a transient/constant behavior for β was achieved. In particular, we selected two distinct regions: a small one (Presidente Prudente) and the biggest region of the São Paulo state (São Paulo city).

**Figure 7:**
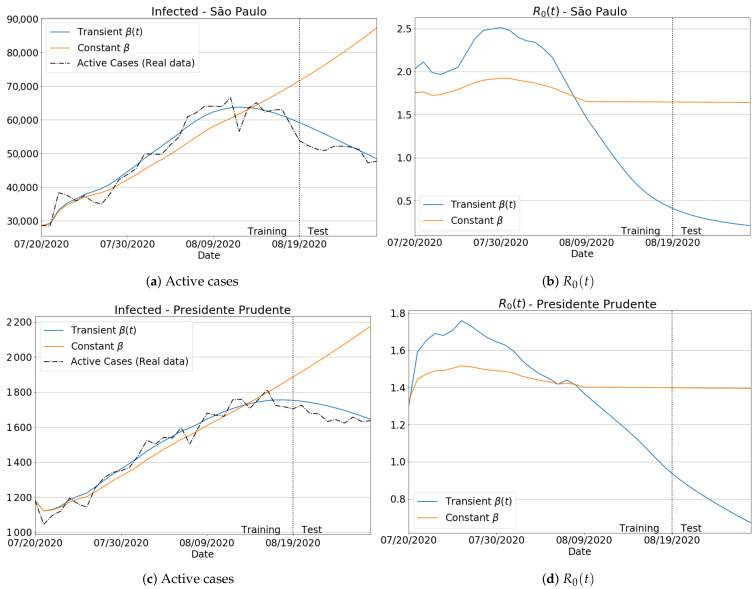
Infected and effective reproduction number using constant and transient values for β: São Paulo region (first row) and Presidente Prudente region (second row).

**Figure 8:**
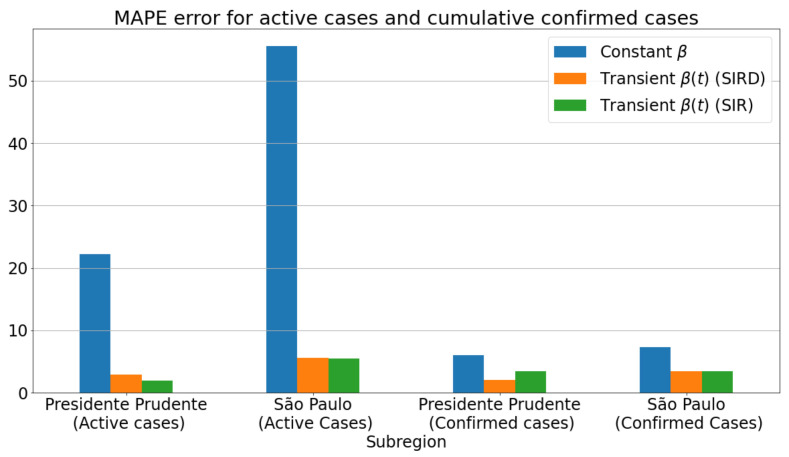
Comparison of MAPE errors for constant and transient values of β: SIR and SIRD models.

Figure 7a,c shows the infected curves. Blue lines give the estimation for transient β, while the orange curves represent the homogeneous behavior for β. From the plotted curves, we can confirm that the best predictions for the number of the infected are those using the $\beta_{net}\left(t\right)$ for both regions. For the effective reproduction number $R_{0}\left(t\right)$, Figure 7b,d displays the importance of taking into account the transient form of β for a more accurate estimation. Notice that by assuming a constant value for β, one can get $R_{0}\left(t\right)$ with a low variation and adjustability, especially in the test period, as no significant changes have been found for the reproduction number. Finally, we measure in Figure 8 the effective impact of transience on β, by assessing the MAPE for the number of infections in the same time period as considered in Figure 7. From the reported scores, one can see that there was a substantial reduction in the MAPE errors as the transmission rate β was estimated from a transient way. Moreover, by assuming a data-driven β learned via an intelligent architecture such as ANN, one can verify that $\beta =\beta_{net}\left(t\right)$ is not only suitable to improve the prediction accuracy of the SIRD-based formulation, but it also improved the well-known SIR model. In fact, both SIR and SIRD when coupled with a learned transmission rate $\beta_{net}\left(t\right)$ performed similarly, producing much lower prediction errors than the case for which β is taken as a non-learned function.

#### 3.3.3. Invariance to Training Periods

**Figure 9:**
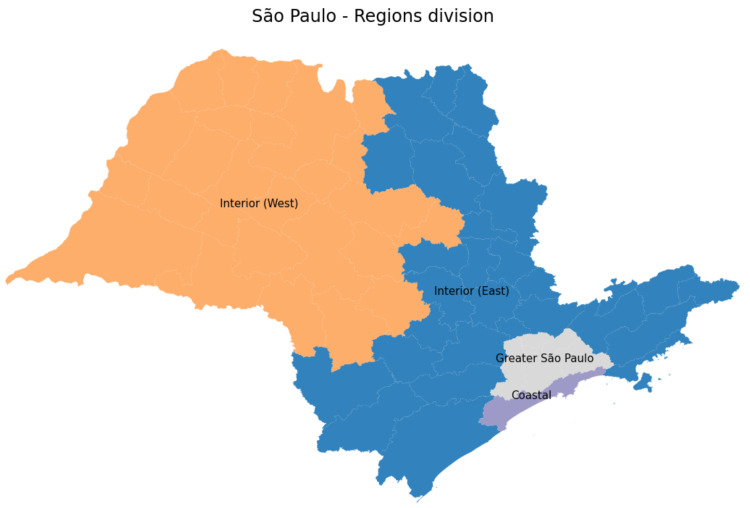
São Paulo state subregions.

This section is dedicated to confirming that our approach can accurately predict accumulated, recovered and deceased cases regardless of the data training period. In order to verify such a method’s feature, we follow the usual subdivision of the São Paulo state to group the whole population into four main regions: *Coastal*, *Greater São Paulo*, *West*, and *East* areas, as illustrated in Figure 9.

As our trained model can estimate distinct epidemiological metrics, in this experiment we focused on the pandemic parameters for which the MAPE can be properly computed, i.e., accumulated, recovered and deceased cases. In our quantitative analysis, we trained our approach by taking a full period of M=30 consecutive days to predict the next 10 days of the aforementioned variables over three different forecasting periods: August, September and October, as listed in Table 3. One can check from the tabulated scores that the predictions were quantitatively accurate and stable since MAPE errors were substantially low for all regions. The maximum MAPE was observed for recovered cases in Greater São Paulo for the first period, while both accumulated and deceased cases delivered low errors, even the biggest measured ones, as reported to death curve of East’s first period (3.465) and Coastal’s second period for Covid-19 cases (1.536). Therefore, our data-driven approach turned out to be stable and robust over different prediction periods, even with a small amount of data taken to generate the training set: a 3-to-1 ratio with respect to the full test set, i.e., 30 past days for training the model, and 10 days for future predictions. In fact, in Table 4, we show that the proposed learning approach still remained unchanged and consistent as the window size of the training set changed.

### 3.4. Quantitative and Qualitative Analyses

**Figure 10:**
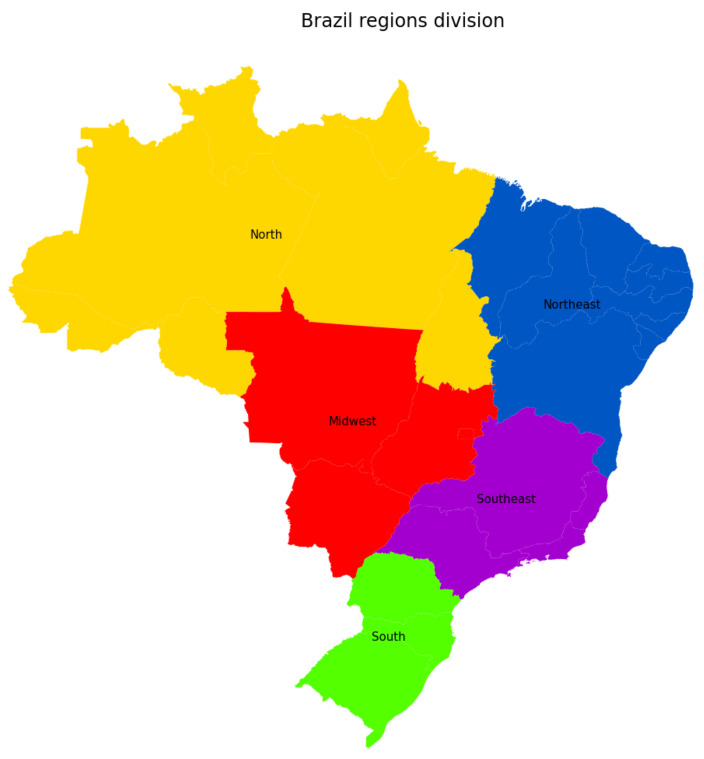
Brazilian regions.

We now discuss the forecasting results provided by the proposed methodology under 10-day time horizons for all the São Paulo regions. Additionally, we extend our analysis to better understand and discuss both the past pandemic situation and the rise of a second wave in the whole country, as we have recently observed from the most current data. In such particular case, the Brazilian dataset has been downloaded directly from the government official source [2], and it covers all the five regions of the country (see Figure 10 for an illustration). From the available data, we performed our analysis in terms of the following Covid-19 indicators: accumulated, recovered and deceased cases. These data, together with new hospitalizations, have been the main pandemic metrics used by the public body to assess the Covid-19 scenario in São Paulo and Brazil [2]. Finally, it is worth mentioning that, in synergy with the efforts made at both state and national levels, we have continuously collaborated with different press conglomerates and public authorities, especially in the last few weeks, where a substantial increase in new cases of Covid-19 and hospitalizations have been firstly warned by our data analysis tool—*Info Tracker* (see [49,50,51] for a few English news published by Brazilian media agencies).

#### 3.4.1. São Paulo State Regions

Firstly, we provide in Table 5 both MAPE and RMSE measurements for the São Paulo state regions. As one can verify, all the MAPE errors were lower than 1, except the Coastal region, where a MAPE of 1.2 was calculated. Regarding RMSE, regions presented very low errors, whose values were on the order of $10^{-2}$ on average, thus attesting to the high-quality performance of our hybrid SIRD enhanced by a machine learning-based approach.

**Figure 11:**
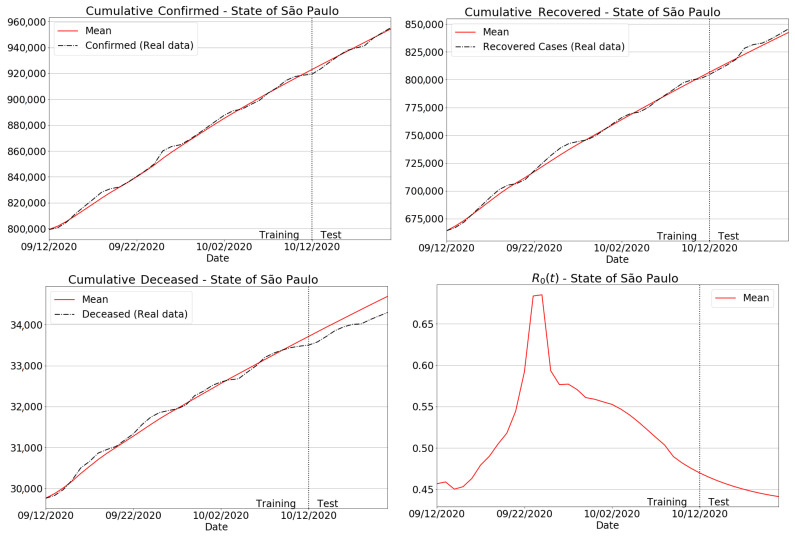
Forecasting results for São Paulo state.

For completeness, we have plotted the results for the São Paulo state in Figure 11. In particular, comparisons between real data and our estimates for accumulated, recovered and deceased cases are presented in the first, second and third columns in Figure 11. To better emphasize the Covid-19 transmissibility in the state, the reproduction number was also displayed in the last column. Considering the prediction intervals from test periods, we can see that the proposed model reached a very accurate agreement between the true data and the forecasts of accumulated, recovered and deceased cases in São Paulo state. Another important aspect to be noted is that our model accurately fits the real data in the training interval, regardless of the epidemiological indicator.

#### 3.4.2. Brazilian Regions

**Figure 12:**
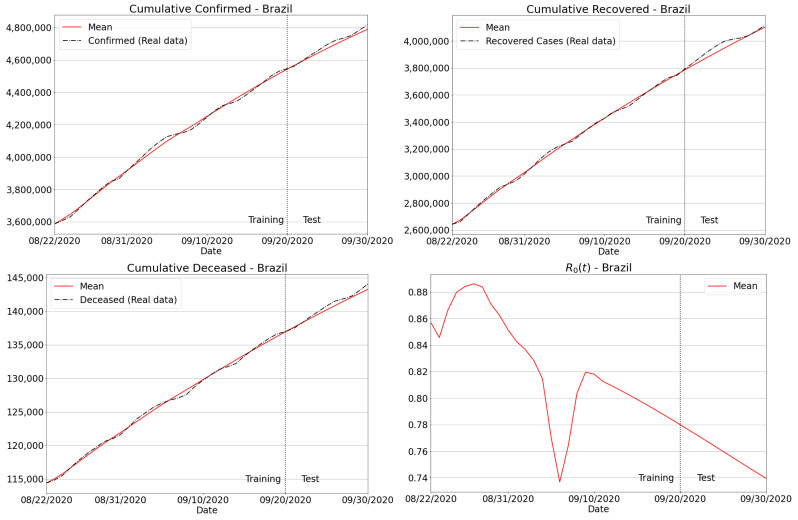
Forecasting results for Brazil.

The next experiment comprised the Brazilian case, where predictions of confirmed, recovered and deceased cases have been delivered for all the five regions of the country. In terms of quantitative assessment, Table 6 reports the MAPE and RMSE, where one can verify that the predictions for the accumulated, recovered and deaths were numerically consistent and reliable. Additionally, we have plotted the results for Brazil in Figure 12, including the predictions of confirmed, recovered and deceased cases. Similar to the São Paulo state case, we also provide $R_{0}\left(t\right)$. Particularly, well-behaved curves were produced considering the extrapolation of real data for the whole country, thus demonstrating the effectiveness of the proposed method in dealing with a huge amount of Covid-19 data.

#### 3.4.3. The Second Wave of Covid-19: Investigations in Brazil and Other Countries

In this section, we discuss our predictions considering the rise of a second wave of coronavirus hitting Brazil. Particularly, one important aspect of our tracking platform is its capability for dealing with real data resulting possibly from a second wave of Covid-19, as already pointed out by our warnings, discussed at the beginning of Section 3.4. Moreover, with the eminence of a fast acceleration in new cases, as reported in European countries in the last months, very recent papers relying on SIR-based models have been presented in the literature (e.g., [52,53,54,55]). Therefore, following the aforementioned works, we make use of our SIRD + machine learning methodology for both purposes: (i) analyzing the historicity of the pandemic’s most recent past in Brazil, and (ii) supporting the state and federal government to implement immediate decisions in order to contain the advance of coronavirus in the country.

**Figure 13:**
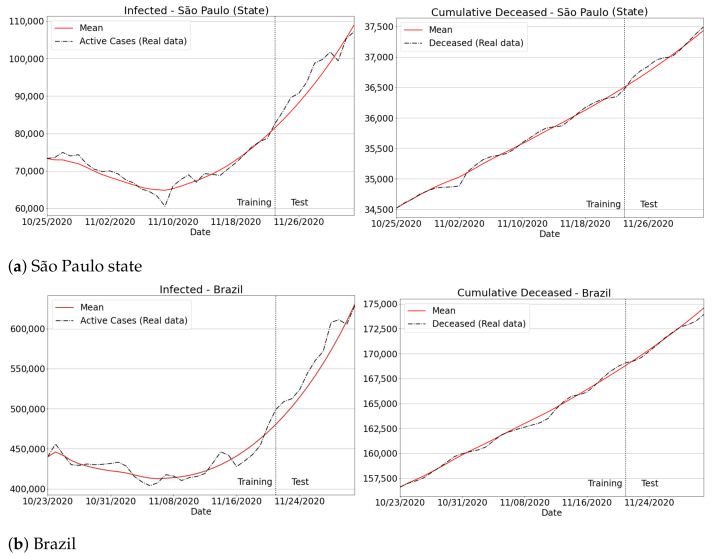
Infected and deaths for São Paulo state and Brazil over recent data. The high increase in both indicators suggests the eminence of a “second wave” of coronavirus hitting the country and starting in the second half of November.

A warning, real case involving the predictions resultant from our approach is depicted in Figure 13. First, one can verify that the trajectory, as well as the real values for new infections and deaths in both training and prediction periods have been successfully captured. Second, the high upward trend of new infection curves suggests that both São Paulo and Brazil have recently suffered from substantial growth in new cases and deaths. Note that the new infections in the state of São Paulo jumped from 70,000 on 14 November to 110,000 on 3 December: an increase of 57% in a short period of 19 days. When inspecting the Brazilian curves, a similar finding was observed: a jump from 415,000, on 14 November, to around 630,000 in early December, i.e., a worrying increment of 51% in just three weeks.

**Figure 14:**
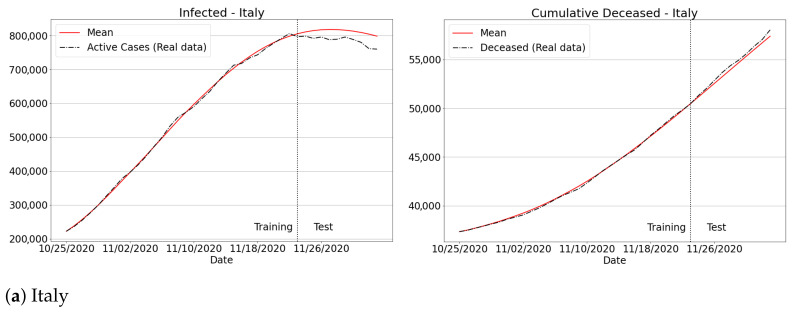

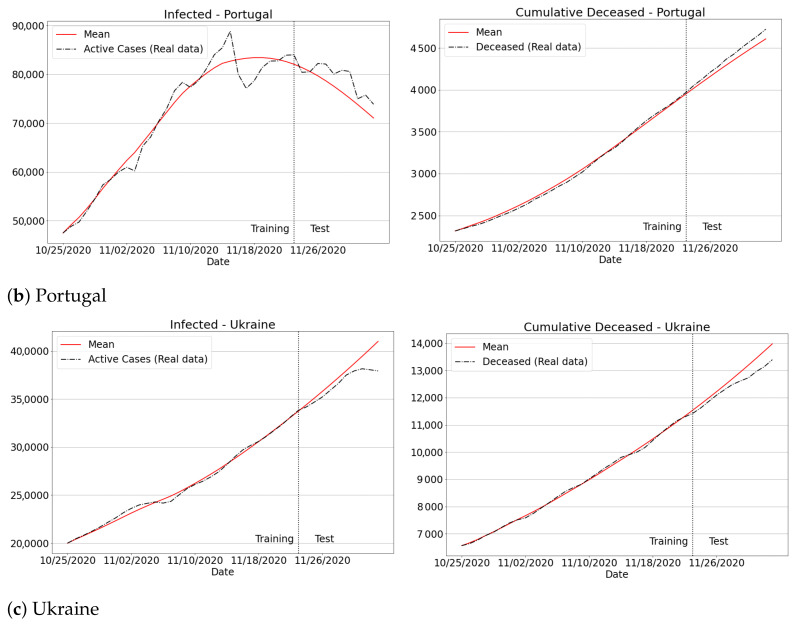
Infected and deaths for Italy, Portugal and Ukraine over recent data.

To provide further evidence concerning the feasibility of the current methodology, we have investigated the spread of Covid-19 for three different data sets: Italy, Portugal and Ukraine. The analysis was conducted considering the data provided by Johns Hopkins University [4], from 25 October to 3 December. Particularly, according to Figure 14, we can observe that our methodology was able to fit the real data for all the European countries concerning the total number of infected and deceased. Therefore, these results confirm that our SIRD model enhanced by a learning scheme can be successfully applied to inspect the Covid-19 spread in several regions of the world.

## 4. Conclusions and Future Work

In this paper we proposed different data-driven responses against the Covid-19 outbreak for São Paulo state and Brazil. These include a free, interactive platform for tracking coronavirus-related data, a novel SIRD-based mathematical model, which learns epidemiological parameters to best fit the corresponding data of each analyzed region, and a comprehensive experimental evaluation of both past and the current situation of the pandemic in Brazil and the state of São Paulo.

As discussed in Section 3.4 and Appendix A, our tracking platform—*Info Tracker*—has supported public authorities, society and press agencies in better understanding and exploiting Covid-19 data, by intuitively interacting with them through a simple and easy-to-communicate interface. Regarding our second contribution against the novel coronavirus, i.e., a functional forecasting model that works properly even when there are delays in case notifications, we have found that the predictions matched the true data both qualitatively and quantitatively. As shown in our battery of tests, our unifying SIRD + machine learning approach produced considerably low MAPE and RMSE errors, as shown in Table 5 and Table 6. Indeed, MAPEs were less than 1 in almost all the measurements. Another important aspect noted in our experiments is that the trained forecaster turned out to be very effective and robust when dealing with badly conditioned data, as shown in Section 3.3.1 (see the listed variances in Table 2).

We discussed in Section 3.4.3 how our predictions can be successfully used to assess the impact of a second Covid-19 wave starting in São Paulo and Brazil, warning about the sudden growth of new cases of coronavirus so as to put health authorities and the country’s population on alert for the coming weeks. Additionally, we have discussed in Section 3.4.3 the applicability of our data-driven model for predicting the Covid-19 spread in Italy, Portugal and Ukraine.

As future work, we plan to incorporate new visualization and interactive features into *Info Tracker* in addition to the study of population mobility between intra-geographical areas as part of our mathematical approach. These are useful, but difficult-to-obtain, data in the Brazilian context, which could improve the modeling of the Covid-19 spread in terms of identifying the spatial–temporal dynamic of the disease flow, similar to [56].

Finally, it is important to point out that several papers are dealing with forecasting of Covid-19 based on learning strategies. In particular, Chen et al. [19] combined Finite Impulse Response (FIR) filter with a ridge regression (regularized least-squares), while in [44] the authors adopted a Genetic Algorithm to estimate the infection rate, delivering a hybrid scheme which combines an ANN and a Fuzzy logic model to forecast Covid-19 data. New studies are welcomed to provide insights concerning the pros and cons of data-driven models. Therefore, as future work, we intend to compare our current methodology with different learning methods to forecast the Covid-19 spread.

## Figures and Tables

**Table 1:** List of symbols.

Notation	Description
S(t)	number of susceptible at time *t*
I(t)	number of infected at time *t*
R(t)	number of recovered at time *t*
D(t)	number of deaths at time *t*
β	transmission rate
β(t)	transient transmission rate
$\gamma_{r}$	rate of recovered
$\gamma_{d}$	rate of mortality
$R_{0}\left(t\right)$ or $R_{t}$	time-dependent reproduction number
$\beta_{net}\left(t\right)$	prediction for the transmission rate at time *t*
*M*	pre-specified training period
*p*	desirable forecast period
$Y_{i}$ and $\dot{Y_{i}}$	real and predicted daily values with respect to a given target variable

**Table 2:** Variance computed during the training process, and average MAPE for active cases (infected) with respect to Figure 6 results.

Region	Variance Norm $\left(||s^{2}|{|}_{2}\right)$	MAPE for Active Cases (%)
Greater São Paulo North	0.098	2.658
Greater São Paulo Southeast	1.478	4.414
Marília	0.378	1.928
Ribeirão Preto	0.063	3.894

**Table 3:** MAPE errors for different forecasting periods.

Region	MAPE Error for Cumulative Cases (%)	MAPE Error for Cumulative Recovered Cases (%)	MAPE Error for Cumulative Deceased Cases (%)
15 August 2020–24 August 2020			
Coastal	1.513	0.951	1.046
Greater São Paulo	0.753	3.731	1.394
Interior (East)	0.454	1.491	3.465
Interior (West)	1.085	1.826	2.618
15 September 2020–24 September 2020			
Coastal	1.536	0.347	2.503
Greater São Paulo	0.598	0.344	0.926
Interior (East)	0.937	0.461	1.157
Interior (West)	1.277	0.753	0.603
15 October 2020–24 October 2020			
Coastal	0.533	0.249	0.268
Greater São Paulo	0.105	0.438	0.776
Interior (East)	1.413	0.886	0.236
Interior (West)	0.832	1.097	0.881

**Table 4:** MAPE errors for Greater São Paulo region as the size of the training window varies.

Training Windows	MAPE Error for Cumulative Cases (%)	MAPE Error for Cumulative Deceases (%)	MAPE Error for Cumulative Recovereies (%)
10-30 days	0.285	0.753	0.293
10-40 days	0.762	0.928	0.321
10-50 days	1.179	0.894	0.592

**Table 5:** Tabulated errors for the predictions depicted in Figure 11 (São Paulo state regions).

Region	Cases		Recoveries		Deaths	
	MAPE	NRMSE	MAPE	NRMSE	MAPE	NRMSE
Costal	0.325	0.004	0.907	0.010	1.200	0.012
Greater São Paulo	0.680	0.007	0.371	0.004	0.714	0.007
Interior (East)	0.818	0.010	0.592	0.007	0.312	0.004
Interior (West)	0.376	0.005	0.626	0.007	0.826	0.009
State of São Paulo	0.219	0.003	0.455	0.005	0.475	0.005

**Table 6:** Tabulated errors with respect to predictions depicted in Figure 12 (Brazilian regions).

Region	Cases		Recoveries		Deaths	
	MAPE	NRMSE	MAPE	NRMSE	MAPE	NRMSE
Midwest	1.169	0.014	0.989	0.013	0.856	0.009
North	0.889	0.010	0.282	0.003	0.173	0.003
Northeast	0.244	0.003	0.342	0.005	0.487	0.005
South	4.413	0.047	7.111	0.072	0.397	0.004
Southeast	0.815	0.009	0.675	0.009	0.427	0.005
Brazil	0.323	0.004	0.638	0.008	0.273	0.003

**Figure A1:**
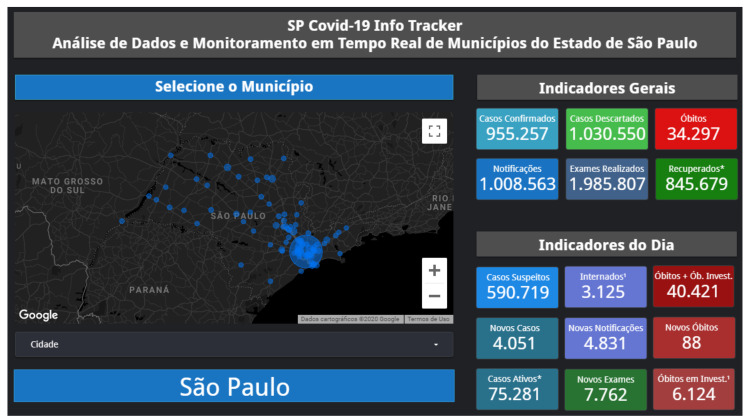
SP Covid-19 Info Tracker Platform (http://www.spcovid.net.br): First page view.

**Figure A2:**
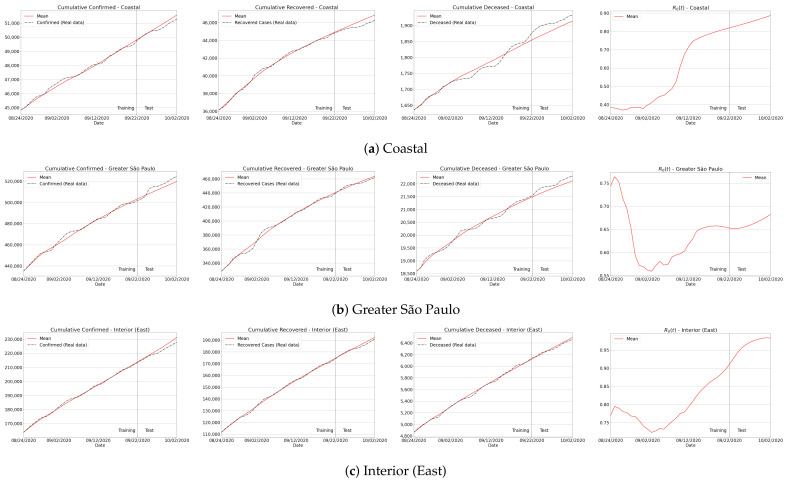

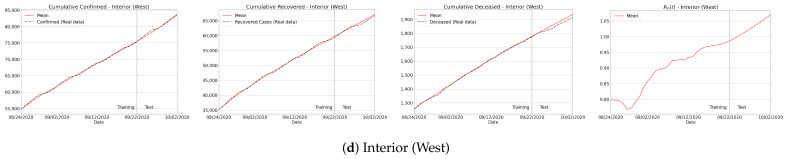
Forecasting results covering all the main regions of São Paulo.

**Figure A3:**
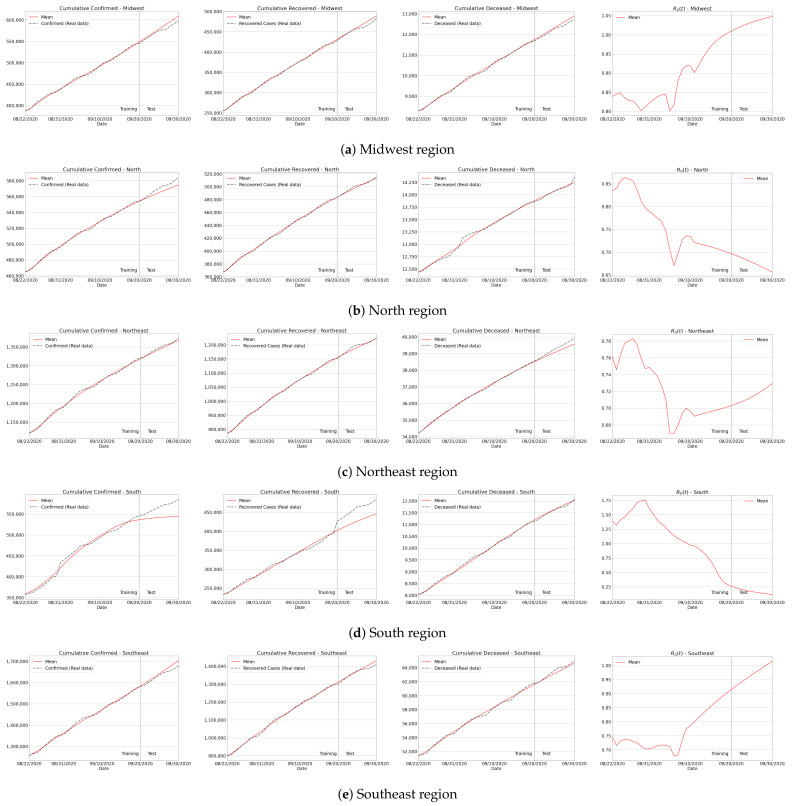
Forecasting results for Brazil’s regions.

## Data Availability

Publicly available datasets were analyzed in this study. This data can be found here: www.spcovid.net.br, https://github.com/CSSEGISandData/COVID-19 [4], and https://covid.saude.gov.br [2].

## Appendix A. SP Covid-19 Info Tracker

Aiming at facing the pandemic scenario in São Paulo state, we designed an interactive platform for real-time monitoring and predictive analysis of Covid-19 data. Our tracking system, called *SP Covid-19 Info Tracker* (Platform website (in Portuguese): http://www.spcovid.net.br), is available to civil society, press agencies, government policymakers and the scientific community, and it provides accurate information and detailed reports about the daily progress of coronavirus in more than 90 cities spread across the state. Figure A1 illustrates the released platform.

The data are collected at the municipal level, i.e., daily taken from the epidemiological bulletins as provided by the 92 city halls monitored by our project [57], which are the primary sources of case notifications in Brazil [37]. In terms of representativeness, these cities together comprise “a universe” of 35 million people, i.e., the same as Poland’s population, or twice as large as the Netherlands’ residents. Another important aspect to be noted is that São Paulo is the most populous state in Brazil, and it has been the epicenter of the pandemic in the country. Until the end of November 2020, the state corresponded to more than 20% of total confirmed cases in Brazil [2].

Our motivation to design a new data repository from first-hand sources comes from the necessity of delivering rapid responses against Covid-19, providing not only more accurate records of new cases and deaths but also the hospitalizations, suspected cases, testing levels, social isolation rates and deaths under investigation, among other pandemic-related indicators that have not been made available by state and federal bodies. Moreover, the use of “fresh” data as promptly published by municipal sources allows us to anticipate the virus spread estimation in the state, thus mitigating the Brazilian government’s delay in updating their reports, as the notifications can take several days or even weeks to be inserted into the central repository, as reported by the Brazilian media [36,37,58]. Once the data are collected, they are made available day after day on the platform, including holidays and weekends. This process has been carried out since 20 March 2020 in an effort to keep the data as accurate as possible.

Similar to the efforts made by the scientific community to overcome Covid-19 in other social contexts of technology use, such as collaborative learning platforms [59] and real-time social-distancing detection systems [60], we focus on promoting digital inclusion through our interactive platform, bringing up coronavirus-related issues like data transparency and epidemiological statistics to those interested in understanding the pandemic’s course in São Paulo and, consequently, in Brazil. The *Info Tracker* platform had over 120,000 visits in 6 months (since June 2020). Finally, our online system has also been used by media agencies to audit municipal governments and other data transparency issues (see the list of news: http://www.spcovid.net.br/notícias).

## Appendix B. Qualitative Results for São Paulo State and Brazilian Regions

As previously observed from the results of São Paulo state (see Figure 11), our predictions can be considered appropriate for Coastal, Greater São Paulo, Interior East and West regions, as depicted in Figure A2. Even in the more drastic case of deaths as shown in the Coastal region, where the reproduction number jumped from 0.5 to 0.9, the method satisfactorily estimates the total number of fatalities. Finally, we present a similar analysis for the five regions of Brazil in Figure A3. From the plotted curves, it can be seen that no matter what kind of signature the reproduction number has (increasing or decreasing, lower or higher 1.0), our approach correctly captured the true data in almost all the cases.

## Appendix C. Algorithm

**Algorithm 1:** Parameter Calibration and Forecast Process
